# Exploring osteosarcoma based on the tumor microenvironment

**DOI:** 10.3389/fimmu.2024.1423194

**Published:** 2024-11-25

**Authors:** Ao Wu, Zhi-kai Yang, Peng Kong, Peng Yu, You-tong Li, Jia-le Xu, Si-shan Bian, Jia-wen Teng

**Affiliations:** ^1^ The First Clinical College of Shandong University of Traditional Chinese Medicine, Jinan, Shandong, China; ^2^ Hand and Foot Orthopaedic Department, Changle County People’s Hospital, Weifang, Shandong, China; ^3^ Department of Minimally Invasive Orthopedics, Affiliated Hospital of Shandong Traditional Chinese Medicine University, Jinan, Shandong, China; ^4^ Department of Traumatology and Orthopaedics, Affiliated Hospital of Shandong University of Traditional Chinese Medicine, Jinan, Shandong, China

**Keywords:** osteosarcoma, tumor microenvironment, immune-related genes, immunotherapy, immunization checkpoints

## Abstract

Osteosarcoma is a cancerous bone tumor that develops from mesenchymal cells and is characterized by early metastasis, easy drug resistance, high disability, and mortality. Immunological characteristics of the tumor microenvironment (TME) have attracted attention for the prognosis and treatment of osteosarcoma, and there is a need to explore a signature with high sensitivity for prognosis. In the present study, a total of 84 samples of osteosarcoma were acquired from the UCSC Xena database, analyzed for immune infiltration and classified into two categories depending on their immune properties, and then screened for DEGs between the two groups and analyzed for enrichment, with the majority of DEGs enriched in the immune domain. To further analyze their immune characteristics, the immune-related genes were obtained from the TIMER database. We performed an intersection analysis to identify immune-related differentially expressed genes (IR-DEGs), which were analyzed using a univariate COX regression, and LASSO analysis was used to obtain the ideal genes to construct the risk model, and to uncover the prognostic distinctions between high-risk scoring group and low-risk scoring group, a survival analysis was conducted. The risk assessment model developed in this study revealed a notable variation in survival analysis outcomes between the high-risk and low-risk scoring groups, and the conclusions reached by the model are consistent with the findings of previous scholars. They also yield meaningful results when analyzing immune checkpoints. The risk assessment model developed in this study is precise and dependable for forecasting outcomes and analyzing characteristics of osteosarcoma.

## 1 Introduction

Osteosarcoma(OS) originates from bone tissue and is a malignant tumor with local invasion and rapid infiltrative metastasis, prevalent in children and adolescents (1, 2). Osteosarcoma has a high disability rate. Currently, surgery combined with chemotherapy is the universal treatment for osteosarcoma, but the survival rate of patients after 5 years is still relatively poor (3). Consequently, there is a pressing requirement to develop novel evaluation methods to enhance the effectiveness of treatment. Immunotherapy has emerged as the most promising treatment in the past few decades.

The immune system plays a role in every phase of tumor formation and advancement. Thus, dysfunction in the immune system plays a significant role in the onset of tumors. When the immune system interplays with the tumor microenvironment, the T cells associated with the anti-tumor immune response will be activated, and they will up-regulate the expression of various inhibitory receptors on their cell surfaces and bind to the corresponding ligands expressed on the exterior of the tumor cells, resulting in the suppression of the immune response, i.e., the intensity of the anti-tumor immune response will be weakened, and ultimately, the tumor cells will be able to achieve immune escape. The goal of immune checkpoint blockade (ICB) therapy is to enhance the functionality of T cells by disrupting the interaction between these receptors and their ligands, thereby enabling more efficient eradication of cancer cells through the immune system. An increasing body of clinical research has shown the efficacy of immune checkpoint blockade (ICB) therapy in the treatment of many kinds of tumor types (4–6). These trials have facilitated the study of the osteosarcoma tumor microenvironment(TME). TME and tumor clinical presentation, prognosis, and response to immunotherapy are closely related (7, 8), and TME is considered a key factor in OS progression (9). Enhanced comprehension of the immune system’s role in osteosarcoma and the tumor microenvironment (TME) contributes to the advancement of immunotherapy for this condition.

In this study, osteosarcoma samples were obtained from an online database and the samples were immuno-scored, divided into two groups, and analyzed for further analysis of immune-related differential genes between the two groups. A comprehensive immune profile was constructed based on the correlation between the expression levels, prognostic value, and immune infiltration levels of these genes. This study may assist in immunological precision therapy.

## 2 Materials and methods

### 2.1 Data acquisition

TARGET-OS fragment per kilobase of transcript per million mapped reads (FPKM) values (https://gdc-hub.s3.us-east-1.amazonaws.com/download/TARGET-OS.htseq_fpkm.tsv.gz) was obtained from UCSC Xena web platform (https://xenabrowser.net/datapages/) for downstream analysis, and Counts values (https://gdc-hub.s3.us-east-1.amazonaws.com/download/TARGET-OS.htseq_counts.tsv.gz) were obtained for differential analysis.

Eighty-eight osteosarcoma samples were initially retrieved from the UCSC Xena web platform; samples with no or incomplete clinical information were excluded, for a total of 84 osteosarcoma samples. The clinical characteristics of 84 patients with osteosarcoma are shown in **Supplementary Table S1**. In addition, we obtained the set of immune-related genes from the TIMER database for subsequent analyses (**Supplementary Table S2**). The somatic mutation data and the copy number variation (CNV) profile were obtained from TCGA (https://portal.gdc.cancer.gov/).

### 2.2 Immune assessment, clustering, and comparison of immune properties

The tumor samples were scored using the ESTIMATE algorithm to obtain StromalScore, ImmuneScore, ESTIMATEScore, and TumorPurity (10), and the samples were divided into two groups, named high and low, based on the average of the above four data sets. Survival analysis was conducted to investigate the correlation between the four parameters and overall survival (OS).

Subsequently, the abundance of 30 immune cells in the tumor samples was assessed using the ssGSEA algorithm (11), and the tumor samples were consistently clustered to obtain immune subtype groupings. An examination was conducted to explore variations in various attributes among clinical phenotypes. Clinical characteristics, including gender, age, survival time, survival status, whether metastatic or not, and previously obtained immune subtype groupings, were visualized using a heatmap, and a box plot was created to assess the levels of immune infiltration among different immune subtypes. In addition, box plots of the four scores obtained from the previous ESTIMATE algorithm were compared according to the immune subtype groupings. To assess the tumor mutational burden (TMB), we examined the total count of unique genes without synonymous somatic mutations per megabase (Mb) in each sample. Truncating mutations comprised frame-shift deletions or insertions, nonsense mutations, and splice-site mutations. In addition, non-truncating mutations encompass in-frame deletions or insertions, missense mutations, and nonstop mutations. We identified mutational differences between the two groups based on immunological grouping. Subsequently, differently expressed genes (DEGs) were identified through comparative analysis of immune subtype classifications utilizing the limma package in R software version 4.3.2, with a significance threshold set at P<0.05 and |logFC|>1. Volcano plots were generated to visualize differentially expressed genes (DEGs), which were further subjected to gene ontology (GO) function annotation and Kyoto Encyclopedia of Genes and Genomes (KEGG) pathway analysis. Pathway differences between immune subtypes were analyzed using GSEA. GO and KEGG analyses were obtained from the DAVID Database (https://david.ncifcrf.gov/) and then visualized using the R software ggplot2 package, and GSEA results were analyzed using the R software clusterProfiler package and the GseaVis package.

### 2.3 Assessment of immune-related DEGs

Immune-related differentially expressed genes (IR-DEGs) were identified by intersecting differentially expressed genes (DEGs) with immune-related genes. The IR-DEGs were imported into the STRING platform for protein-protein interaction (PPI) analysis, and the IR-DEGs were analyzed for their functions using GO functional annotation and KEGG pathway analysis. The intersecting genes were also analyzed for GSEA enrichment using the above methods. Finally, the MCC algorithm in Cytoscape software version 3.10.1 was used to obtain the top ten genes. Next, we explored the overall survival and immune infiltration among various immune subtypes based on the top ten genes.

### 2.4 Risk model construction

By setting the significance level at P < 0.05, the univariate Cox regression analysis was conducted to explore the IR-DEGs and identify genes related to survival outcomes. Next, the study utilized Least Absolute Shrinkage and Selection Operator (LASSO) estimation for survival modeling of genes showing significant correlations with survival to identify potential candidate genes. Following this, the sample’s risk score was computed based on the selected candidate gene. The samples are divided into training sets and test sets on average. The grouping is strictly randomly followed, and there is no statistical difference between the two groups. Calculate the prognosis of the training set, test set, and all groups, and present with the ROC curve.

### 2.5 Prognosis of features

We first assessed tumor immune escape and immune checkpoint blockade responses using the Tumor Immune Dysfunction and Exclusion (TIDE) online website (http://tide.dfci.harvard.edu/), followed by risk scoring to divide the sample into a high-risk scoring group and a low-risk scoring group, and then TIDE values were calculated between the two groups, with higher TIDE scores associated with poorer immune checkpoint blockade therapy. The differences in immune checkpoint-related genes between the two groups were subsequently calculated and represented by a scatter plot. The samples were categorized based on the high and low levels of candidate genes, and then the disparity in immune checkpoint-related genes was computed between the two sets. This dissimilarity was visually depicted through a scatter plot to investigate the potential connection between the candidate genes and immune checkpoint genes. The immune infiltration of all samples was calculated using CIBERSORT to screen for differential immune cells between the high-risk scoring group and the low-risk scoring group, and the expression of candidate genes in the differential immune cells was calculated. MicroenvironmentScore was calculated for all samples using the xCell (https://xcell.ucsf.edu/) online site. Differences in MicroenvironmentScore between the high-risk scoring group and the low-risk scoring group were calculated and visualized in a boxplot.

## 3 Results

### 3.1 A holistic landscape of immunological features

**Figure 1:**
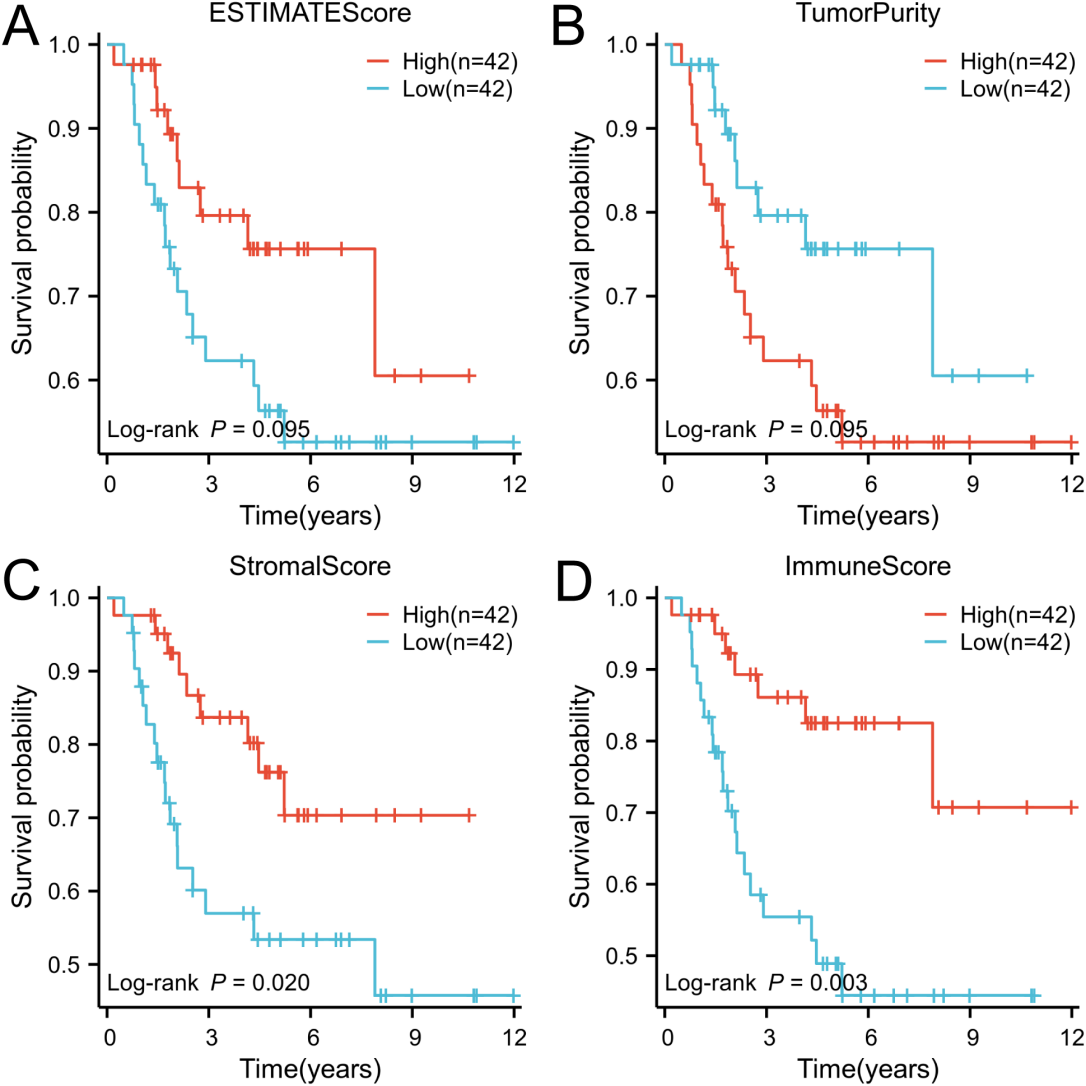
The ESTIMATE algorithm was utilized to conduct survival analysis on patient groups categorized based on high and low immune scores, and the results were visually represented through Kaplan-Meier (K-M) curves. **(A)** ESTIMATE score, **(B)** Tumor purity score, **(C)** Stromal score, and **(D)** Immune score.

Based on the tumor stroma and immune characteristics of osteosarcoma, the acquired osteosarcoma samples were analyzed using the ESTIMATE algorithm to reveal the level of immune infiltration of tumor samples in osteosarcoma. StromalScore, ImmuneScore, ESTIMATEScore, and TumorPurity were calculated and analyzed in the tumor samples (**Supplementary Table S3**). The scores were grouped according to their median (median of StromalScore, ImmuneScore, ESTIMATEScore, and TumorPurity were 488.57, 424.86, 1095.16, and 0.72 in that order), and were named as high and low groupings, respectively. These scores reflect the different compositions of the tumor microenvironment, assessing the degree of stromal and immune cell infiltration, and by grouping them, it is possible to understand the biology of the tumor better and to explore its relationship with tumor prognosis by performing a Kaplan-Meier curve analysis based on survival time and status (**Figures 1A–D**). In StromalScore, ImmuneScore, and ESTIMATEScore, high grouping was significantly associated with high survival, while low TumorPurity was associated with high survival. Of these, ImmuneScore was most significantly associated with survival. The data obtained from the above suggests that osteosarcoma can be analyzed prognostically based on ImmuneScore, followed by ssGSEA analysis.

**Figure 2:**
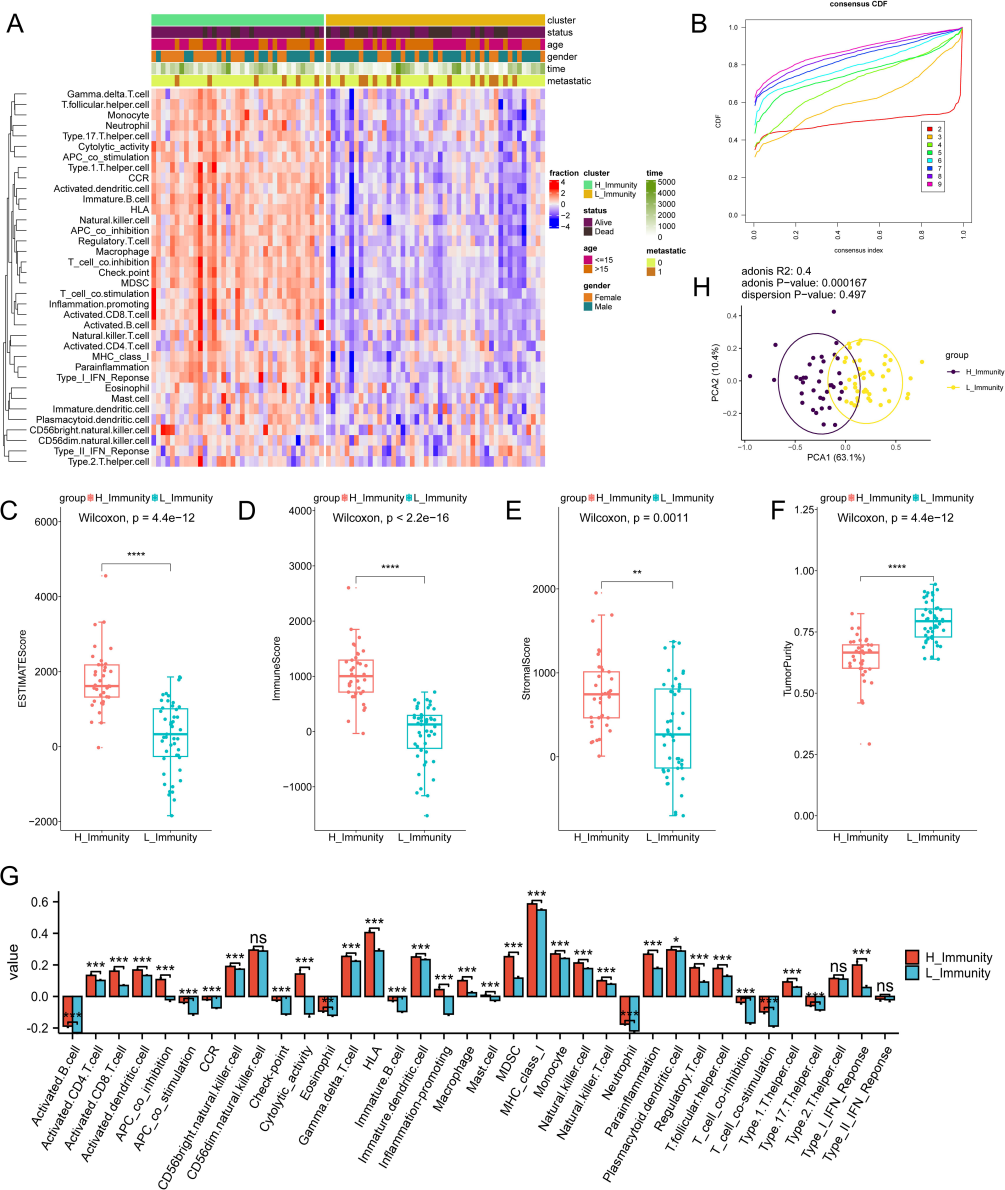
Immune subtype identification and comparative analysis. The symbols * represent p-values less than 0.05, ** represent p-values less than 0.01, *** represent p-values less than 0.001, **** represent p-values less than 0.0001. **(A)** ssGSEA analyses of 84 samples, divided into two groups based on 30 ssGSEA scores per sample. **(B)** Unsupervised clustering of the samples based on their immunological characteristics, where the number of clusters with the highest average within-group agreement is 2. **(C–F)** shows, in order, the differences in ESTIMATEScore, ImmuneScore, StromalScore, and TumorPurity between the high and low immunity groups. **(G)** A box plot is utilized to display the levels of immune cell infiltration in groups categorized as either having high or low immunity. In this visualization, the red boxes correspond to the high immunity group, while the blue boxes correspond to the low immunity group. **(H)** PCA analysis of the two immune subtypes, with purple and yellow dots representing the immunity–high and —low groups, respectively. "ns" stands for no statistical difference.

The impact of immunity on osteosarcoma was explored using ssGSEA (**Supplementary Table S4**). Subsequently, unsupervised clustering was performed on the ssGSEA results, and the number of clusters with the highest average within-group concordance was 2 (**Figure 2B**). Therefore, based on their immune characteristics, the samples were categorized into two groups, and the 30 immune cells were subsequently displayed along with clinical characteristics including gender, age, survival time, survival status, and whether the tumor has metastasized or not (**Figure 2A**). There are noticeable variations in immunological features observed between the clustered groups. Groups with rich immune profiles were named high immune groups, therefore, groups with lower immune profiles were named low immune groups (**Supplementary Table S5**). Tumour metastasis was more frequent in the low-immunity group than in the high-immunity group. To further investigate the relationship between immune groupings and the levels of TME, immune activation, and tumor cell infiltration, several analyses of osteosarcoma TME were performed. As expected, StromalScore, ImmuneScore, and ESTIMATEScore were higher and TumorPurity was lower in the hyperimmune group (**Figures 2C–F**), suggesting that the hyperimmune group was associated with higher survival. Immune checkpoints and immune cell abundance were also significantly higher in the hyperimmune group (**Figure 2G**). Subsequent Principal Component Analysis (PCA) was performed and the immune characteristics differed significantly between the two groups (**Figure 2H**), therefore, we hypothesized that the immune groupings found above could well distinguish the immune and genetic characteristics of the samples.

### 3.2 Mutation analysis

**Figure 3:**
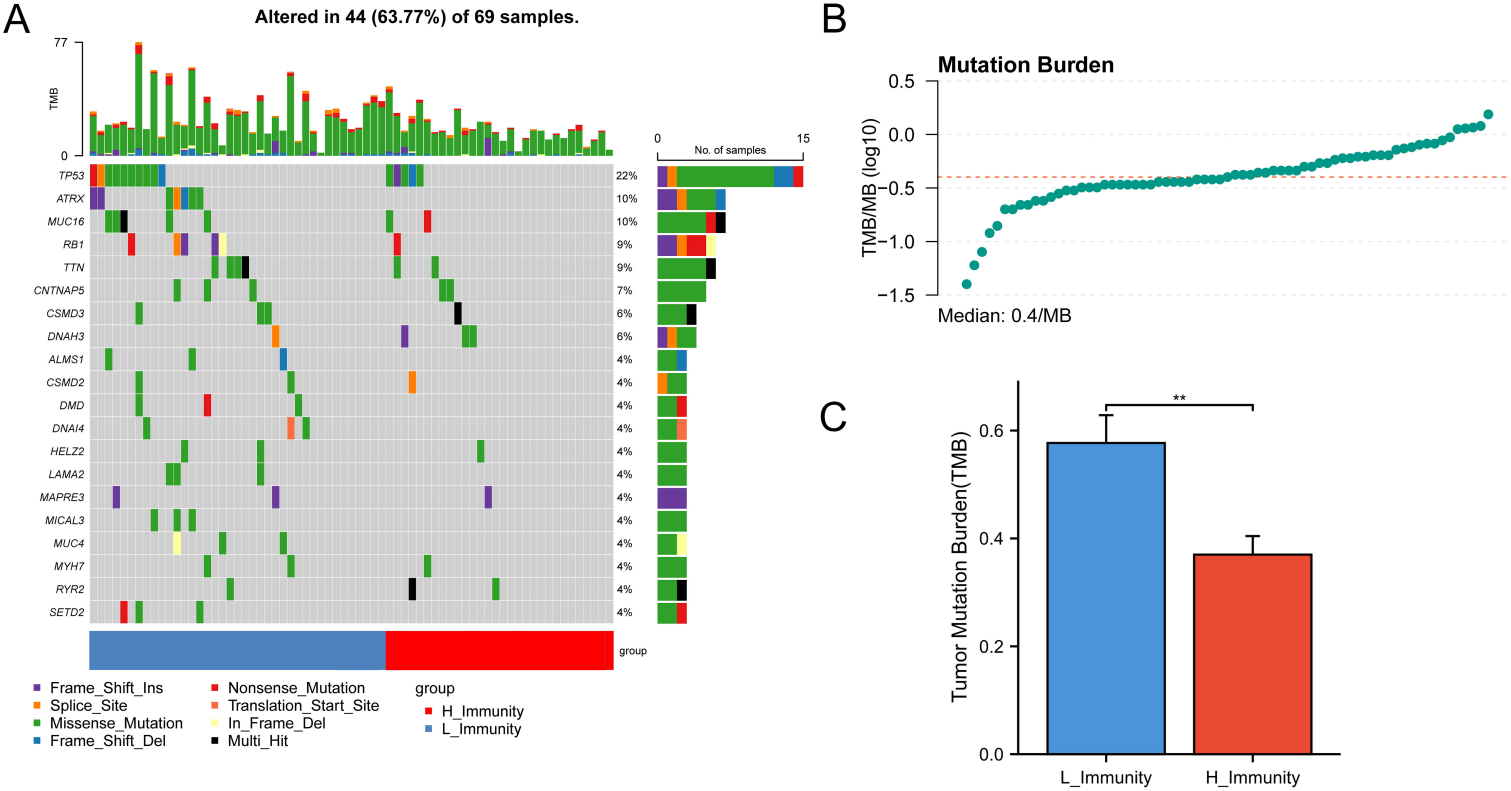
Mutations between high and low immunity groups. The symbols represent p-values less than 0.01. **(A)** Mutation status of genes in the high and low immunity groups. **(B)** TMB distribution of all samples. **(C)** Bar graph showing the difference in TMB between the high and low immunity groups. ** represent p-values less than 0.01.

The analysis of gene mutations revealed a notably elevated mutation rate in the low immunity group, with TP53 being identified as the gene exhibiting the highest frequency of mutations (**Figure 3A**). Tumor Mutation Burden (TMB) analysis was carried out (**Supplementary Table S6**) and visualized using scatter plots (**Figure 3B**). Following this, an analysis of TMB in the high and low-immunity cohorts demonstrated a variation in TMB levels between the two groups, with TMB showing an elevation in the low-immunity cohort (**Figure 3C**). We hypothesized that this may be related to the poorer prognosis of the low-immunity group.

### 3.3 Screening and evaluation of differentially expressed genes

**Figure 4:**
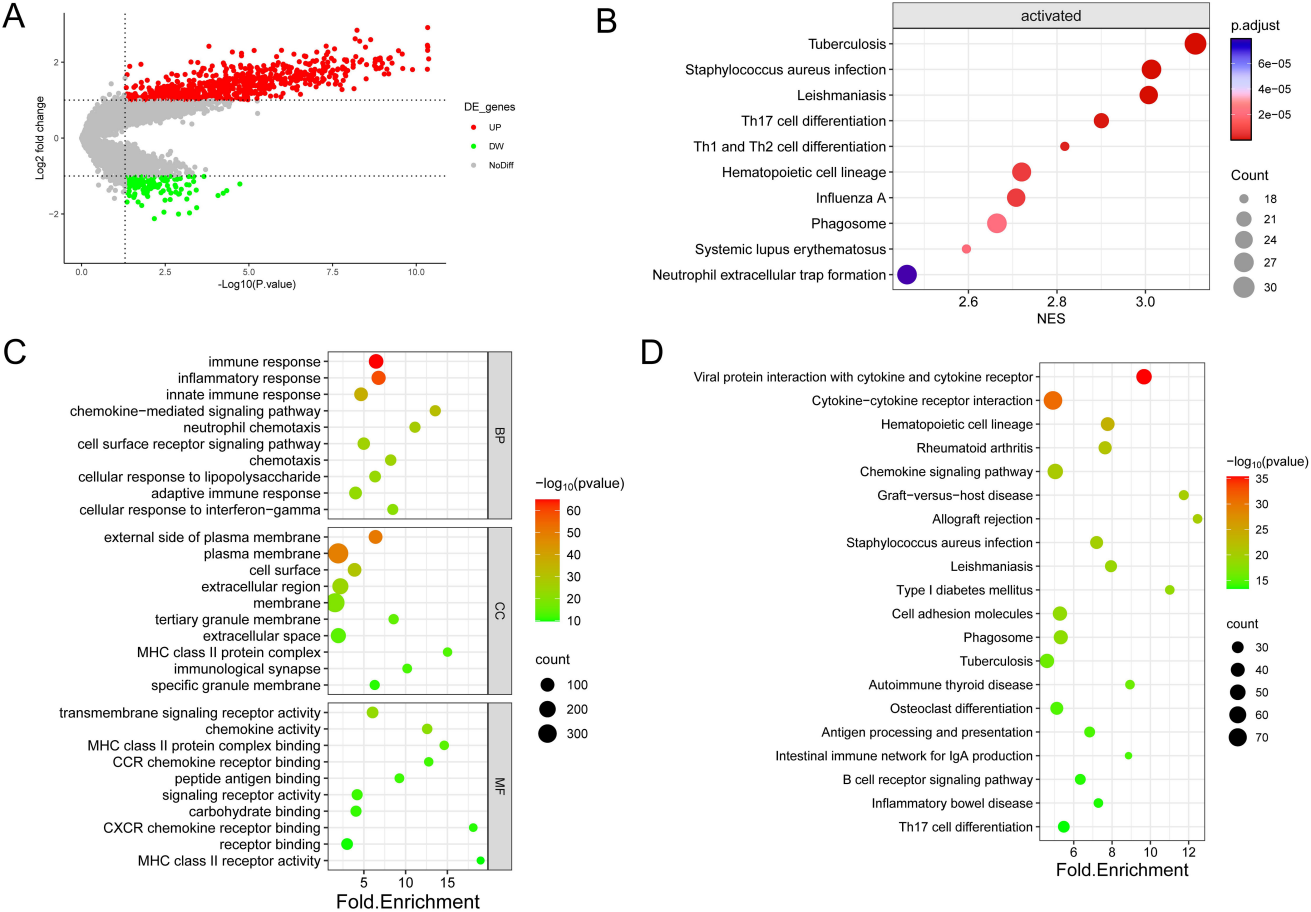
Enrichment analysis was conducted on the DEGs identified in the two immune subtypes. **(A)** Volcano diagram showing the regulation of DEG expression, with green, grey, and red dots representing down-regulation, unregulation, and up-regulation, respectively. **(B)** Bubble diagram showing the top ten pathways according to Gene Set Enrichment Analysis (GSEA). **(C)** Bubble plots showing the top 10 enriched GO BP, CC, and MF. **(D)** Bubble plots showing the top 20 enriched terms of the KEGG pathway, with the size of the dots representing the number of enrichments.

The examination of gene expression variations between immune subtypes identified a pool of 836 differentially expressed genes for subsequent scrutiny (**Supplementary Table S7**), with 697 genes showing up-regulation (83.37%) and 139 genes demonstrating down-regulation (16.63%) (**Figure 4A**). Enrichment analyses of DEGs by GO annotation and KEGG pathway enrichment analyses identified 497 biological processes (BPs), 94 cell components (CCs), 118 molecular functions (MFs), and 69 KEGG pathways (**Figures 4C, D**). The DEGs were also analyzed for GSEA enrichment (**Figure 4B**).

As shown in the figure, the top ten biological processes were filtered according to P-value, most of which were related to immunity, including immune response, inflammatory response, innate immune response, neutrophil chemotaxis, and adaptive immune response. In addition, KEGG pathways are also related to immunity, including Phagosome, Antigen processing and presentation, B cell receptor signaling pathway Th17 cell differentiation, etc. In summary, DEGs and immunity are closely related, subsequently, GSEA enrichment analysis of DEGs was performed to further investigate the pathway differences between immune subtypes, based on P<0.05, a total of 47 enriched pathways were obtained, and the top ten were Tuberculosis (NES=3.11,p.value=6.73E-10) in order, Leishmaniasis (NES=3.01,p.value=3.03E-09), Staphylococcus aureus infection (NES=3.01,p.value=7.05E-09), Th17 cell differentiation (NES=2.90,p.value=4.69E-08), Th1 and Th2 cell differentiation (NES=2.82,p.value=1.13E-07), Hematopoietic cell lineage (NES=2.72,p.value=5.33E-07), Influenza A (NES=2.71,p.value=4.87E-07), Systemic lupus erythematosus (NES=2.60,p.value=1.83E-06), Phagosome (NES=2.66,p.value=2.21E-06) and Neutrophil extracellular trap formation (NES=2.46,p.value=9.22E-06). These findings imply that the activation of the immune system in the tumor microenvironment is implicated in the development of osteosarcoma.

### 3.4 Screening and Evaluation of IR-DEGs

**Figure 5:**
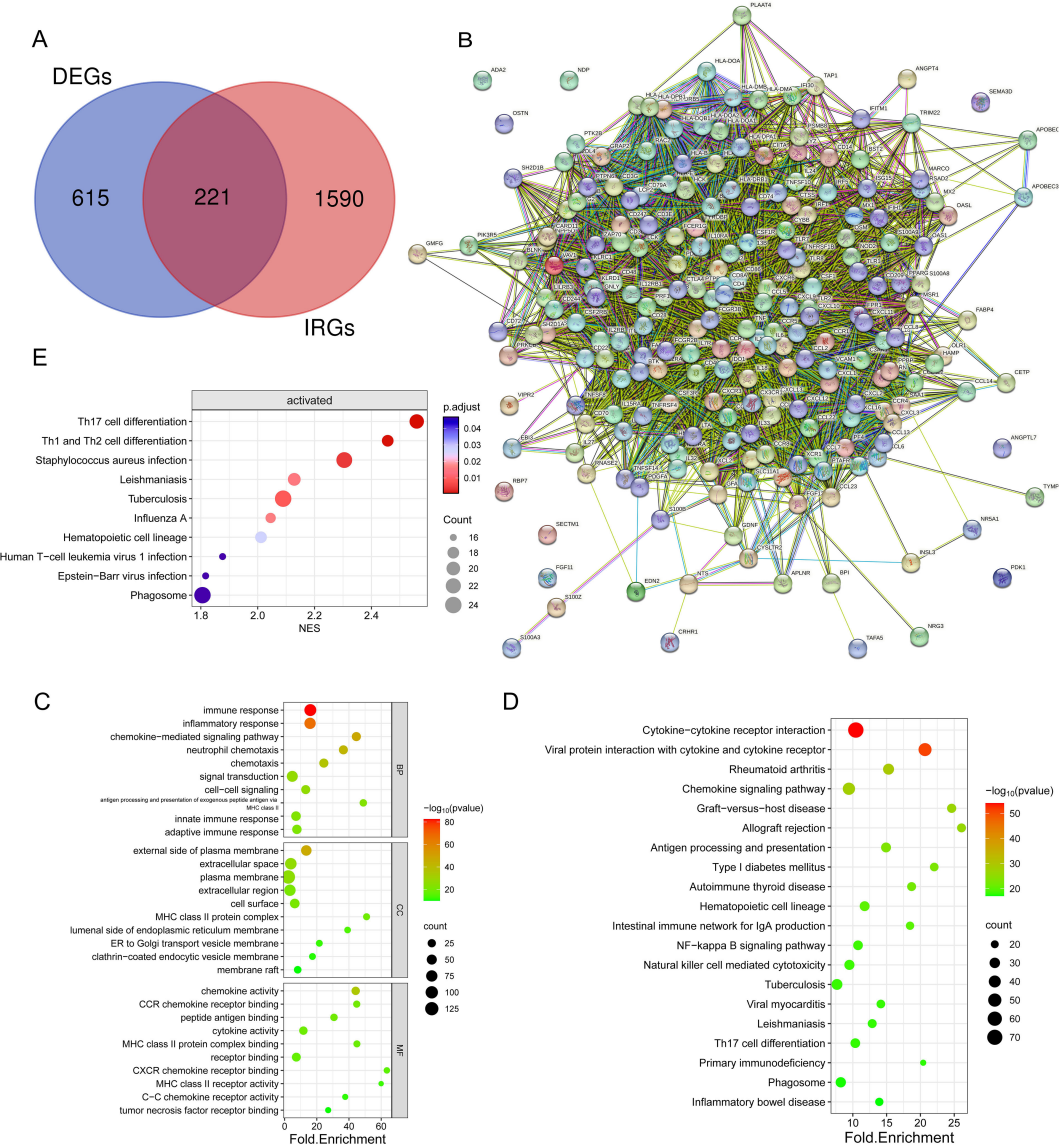
Identification and enrichment analysis of differentially expressed genes (DEGs) associated with the immune system. **(A)** Venn diagram showing 221 immune-associated DEGs overlapping 836 DEGs and 1811 IRGs. **(B)** Protein interaction network diagram of DEGs **(C)** Bubble plots showing the top 10 enriched GO BP, CC, and MF. **(D)** Bubble plots showing the top 20 enriched terms of the KEGG pathway, with the size of the dots representing the number of enrichments. **(E)** Bubble plots showing the top 20 enriched terms of the KEGG pathway, based on GSEA analyses of the top 10 pathways with the highest gene enrichment.

From the TIMER database, 1811 immune-related genes were obtained, and these genes intersected with DEGs to obtain 221 IR-DEGs (**Figure 5A**). The IR-DEGs were entered into the STRING online database to obtain the protein-protein interaction network (**Figure 5B**). The IR-DEGs were subjected to GO annotation and KEGG pathway enrichment analyses (**Figures 5C, D**), resulting in 418 BP, 57 CC, 57 MF, and 69 KEGG pathways. These IR-DEGs were mainly enriched in biological pathways such as immune response, inflammatory response, and adaptive immune response. Among KEGG-enriched pathways, the top five were Cytokine-cytokine receptor interaction, Viral protein interaction with cytokine and cytokine receptor, Rheumatoid arthritis, Chemokine signaling pathway and Graft-versus-host disease. GSEA enrichment analyses were also performed for IR-DEGs (**Figure 5E**). Their top five were Th17 cell differentiation (NES=2.56, p.value=1.35E-05), Th1 and Th2 cell differentiation (NES=2.46,p.value=3.33E-05), Staphylococcus aureus infection (NES=2.30,p.value=0.0002), Tuberculosis (NES=2.09,p.value=0.0005), Leishmaniasis (NES=2.13,p.value=0.001). Similar to the above results. According to the results, it is clear that immune activation, especially T cells, is important for the development of osteosarcoma.

Following that, an examination of the connections and relationships among these IR-DEGs was carried out through the analysis of the protein-protein interaction network (**Supplementary Table S8**), and associations were found for a variety of IR-DEGs, with the most significant correlations between IL6, IL10, CD4, CD8A, IL1B, TNF, and CCL5 and the other immune IR-DEGs.

**Figure 6:**
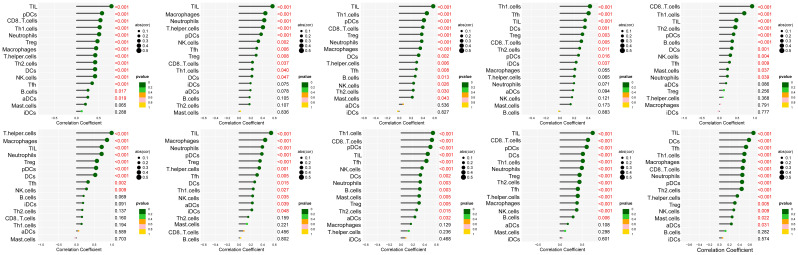
Analysis of immune cell infiltration was conducted on the ten hub genes, visualized using lollipop charts.From left to right, the order is CCR5, TNF, IL10, IL6, CD8A, CD4, IL1B, CCR7, CCL5, and CCL2.

**Figure 7:**
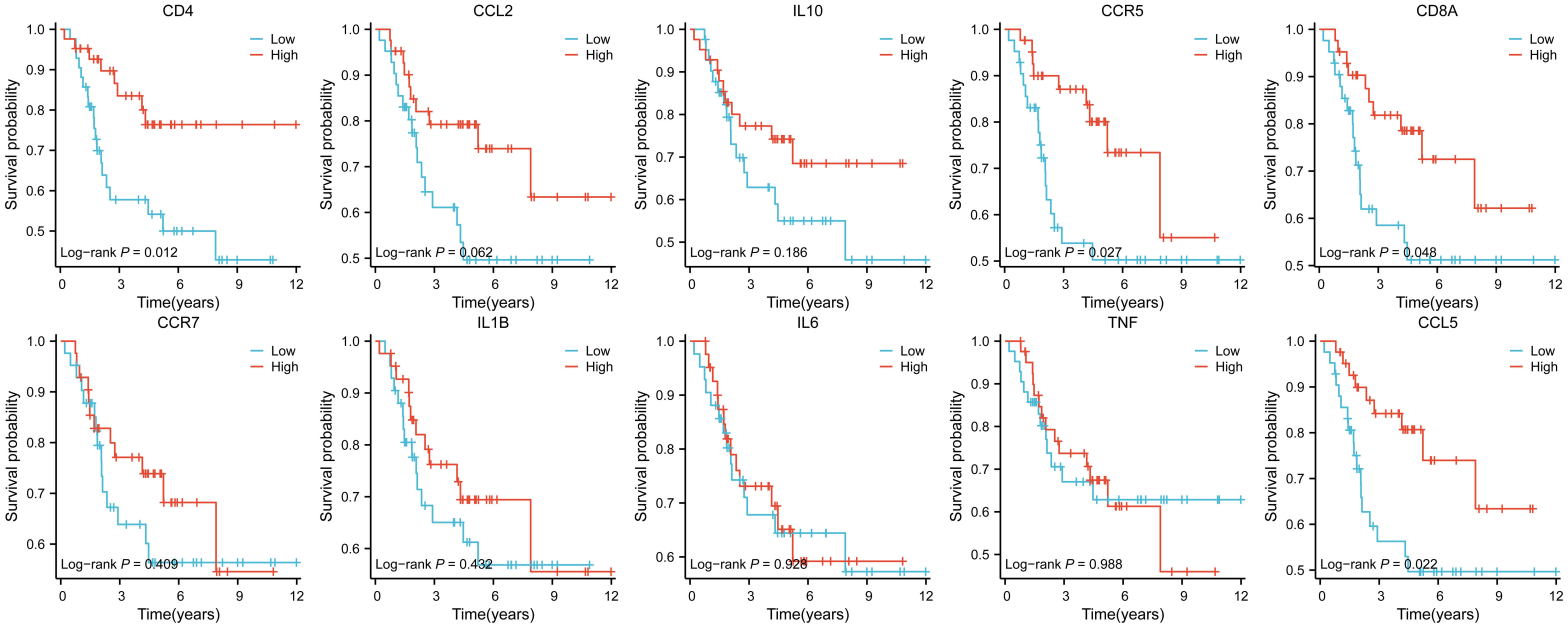
Ten hub genes were subjected to survival analysis.

The interaction network is characterized by the presence of Interleukin (IL) family genes (including IL10, IL1B and IL6), T-Cell Surface Glycoprotein genes (including CD8A, CD86 and CD4), C-C Motif Chemokine Receptor genes (including CCR7 and CCR5) and C-C Motif Chemokine Ligand genes (including CCL2 and CCL5), which are among the hub nodes. Subsequently, the MCC algorithm was applied to obtain the top ten genes (**Supplementary Table S9**), and prognostic survival analysis and immune infiltration analysis were performed on these ten hub genes (**Figures 6**, **7**).

The results showed that ten genes were closely associated with immune cells, and among all hub genes, Tumor Infiltrating Lymphocyte (TIL) infiltration levels were the highest, and mast cells and dendritic cell infiltration levels were lower. In the K-M survival analysis of the hub genes, CD4, CD8A, CCR5, and CCL5 were prognostically significant.

### 3.5 Risk modelling

**Figure 8:**
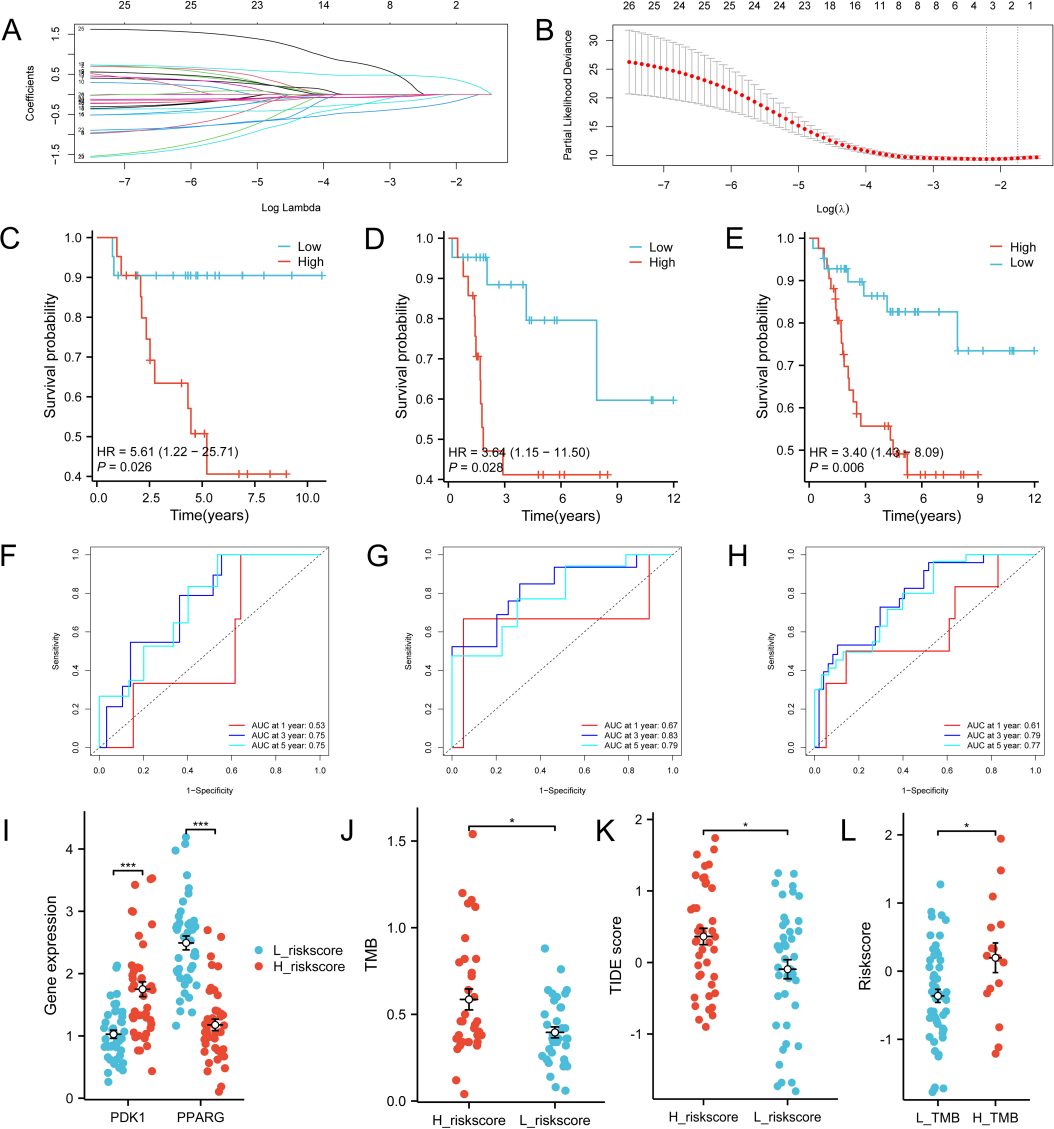
Construction of the total risk profile. The symbols * represent p-values less than 0.05, *** represent p-values less than 0.001. **(A, B)** Employing the LASSO method for the identification of key candidate genes. **(C–E)** Survival analysis K-M curves for the training cohort, validation cohort, and initial combined cohort, respectively. **(F–H)** ROC curves were generated to assess the prognostic significance of risk features in the training, validation, and original merged groups. **(I)** Differences in PDK1 and PPARG gene expression between high and low-risk score groups. **(J)** Differences in TMB between high and low-risk score groups. **(K)** Differences in TIDEscore in high and low-risk score groups. **(L)** Difference in risk scores between high and low TMB groups.

Twenty-six genes were screened from 221 IR-DEGs using one-way COX analysis based on p<0.05, and then the 26 genes were further analyzed by applying the LASSO algorithm using ten-fold cross-validation when lambda.1se= 0.1745826 (**Figures 8A, B**), a risk model was developed using two candidate genes, namely PDK1 and PPARG. Utilizing the expression levels and coefficient values of the candidate genes, a risk score model was created through the application of the subsequent formula: risk score = (0.6839441 × PDK1 expression) + (-0.6420120 × PPARG expression). The risk score formula was utilized on all samples to calculate the risk score for each sample (**Supplementary Table S10**). Subsequently, The samples were divided into two cohorts at random, with no statistical distinction observed between the pair. The median risk scores of the original dataset, the training set, and the test set were used as thresholds (-0.279689391, -0.2077641135, and -0.3399543645), which were divided into a high-risk score group and a low-risk score group, respectively. To assess the accuracy of the risk scoring model constructed by PDK1 and PPARG on prognosis and to provide effective biomarkers for the prediction of osteosarcoma. K-M curve analysis and ROC curve analysis were then performed on the two cohorts and the original combined cohort (**Figures 8C–H**). ROC curves with area under the curve (AUC) values greater than 0.5 were considered statistically different. The results showed that the p-value of the K-M curve was less than 0.05 for the training set, the test set, and the merged set, so this feature was considered to have prognostic value. Whereas, in all three cohorts, the ROC curves indicated that the AUC values for 1-year, 3-year, and 5-year were above 0.5, and the feature had a higher predictive sensitivity for 3 and 5 years (AUC values were greater than 0.7 for both 3 and 5 years). In all three cohorts, a higher survival advantage was demonstrated for low-risk scores, and low-risk scores were strongly correlated with high survival.PDK1 and PPARG were analyzed according to the risk score grouping, and it was found that the expression of PDK1 was positively correlated with the risk score, while the expression of PPARG was inversely correlated with the risk score (**Figure 8I**). Then, the differences in TMB between the high and low-risk score groups were compared based on the previously calculated TMB values, and as expected, there was a difference between the two groups (P<0.05) (**Figure 8J**), with higher TMB values in the high-risk score group, which may be related to the poorer prognosis of the high-risk score group. Subsequently, we calculated the TIDE scores of the tumor samples (**Figure 8K**), which were higher in the high-risk scoring group, suggesting that immune checkpoint blockade therapies were less effective in the high-risk scoring group. In contrast, the opposite was true for the low-risk scoring group. Finally, we used the third quartile of the TMB(0.62 muts/Mb) as a threshold to classify the TMB into two groups: high and low scores. The top 25% of patients were defined as the high TMB group, and the results, as shown in **Figure 8L**, showed that the high TMB group had a worse prognosis.

### 3.6 Performance of risk models

**Figure 9:**
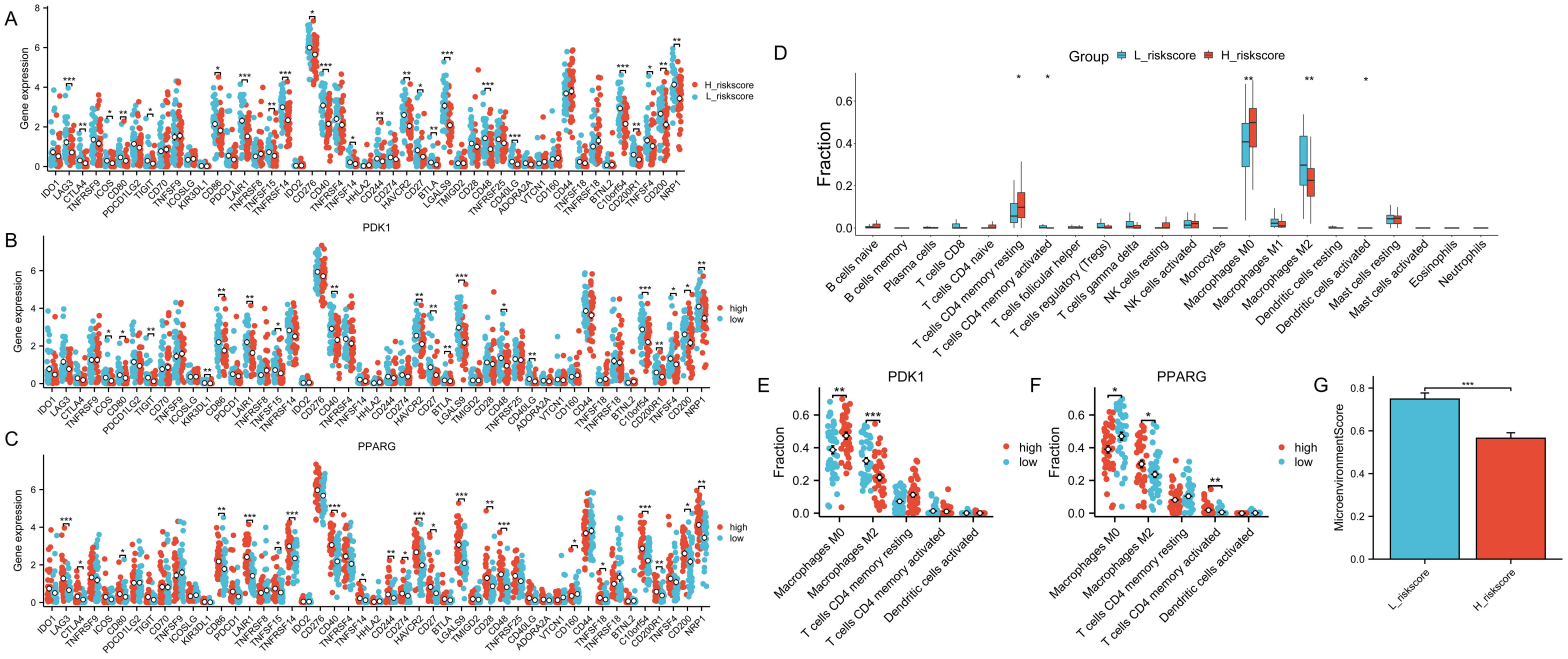
Role of risk models for immune checkpoints. The symbols * represent p-values less than 0.05, ** represent p-values less than 0.01, and *** represent p-values less than 0.001. **(A)** Comparison of the expression levels of immune checkpoint-related genes in the group with high-risk scores and the group with low-risk scores. **(B, C)** The expression levels of immune checkpoint-related genes between the two groups by dividing the gene expression of PDK1 and PPARG into high and low groups according to the median. **(D)** CIBERSORT immune infiltration analysis of all samples. **(E, F)** Infiltration abundance levels of some immune cells by dividing gene expression of PDK1 and PPARG into high and low groups by median. **(G)** MicroenvironmentScore in the high and low-risk scoring groups.

The samples were categorized based on the median risk score(-0.279689391), resulting in the formation of a high-risk group and a low-risk group. Subsequently, the variations in immune checkpoint-associated genes between these two groups were analyzed (**Figure 9A**). The results showed that most of the immune checkpoint-associated genes were differentially expressed, and of the genes that were differentially expressed, all had higher gene expression values in the low-risk score group. Differences in the expression of immune checkpoint-related genes in the high-risk scoring group and the low-risk scoring group are shown in **Supplementary Table S11**. Subsequently, to assess the correlation of immune checkpoint-associated genes with PDK1 and PPARG, the two candidate genes were grouped according to the median log2(FPKM+1) value (PDK1: 1.24; PPARG: 1.75) into high and low expression groups, with differences in the expression of most of the genes (**Figures 9B, C**). High expression of PDK1 was positively correlated with low expression of immune-related genes, while high expression of PPARG was positively correlated with high expression of immune-related genes. The two candidate genes were shown to have opposite roles. Subsequently, CIBERSORT immune infiltration analysis was performed (**Figure 9D**), which showed differences in five immune cell subpopulations. Subsequently, the two candidate genes were still grouped according to median expression, and differential analysis of the five immune cell subpopulations obtained by CIBERSORT immune infiltration showed that high expression of PDK1 was positively correlated with high expression in Macrophages M0 and inversely correlated with high expression in Macrophages M2, and the opposite was true in PPARG (**Figures 9E, F**). Once again, the two candidate genes were shown to have opposite roles. Finally, the samples were analyzed using the xCell online website to obtain the MicroenvironmentScore (**Supplementary Table S12**), which was significantly different between the high-risk scoring and low-risk scoring groups and was higher in the low-risk scoring group (**Figure 9G**).

## 4 Discussion

Recent studies have shown that cancer development is influenced by the activation of the immune response (12). To delve deeper into the immune-related mechanisms of osteosarcoma, we explored the DEGs across two groups of immune characteristics. Furthermore, the majority of the IR-DEGs were found to be up-regulated in our study, exhibiting substantial enrichment in various immune-related biological pathways. Macrophages play a significant role in the tumor microenvironment, and in tumor development, tumor-associated macrophages can interact with other immune cells in the tumor to promote tumor development and progression. In addition, they can suppress tumor growth by promoting the phagocytosis of the cells (13). Furthermore, it has been shown that cytotoxicity of T cells can lead to tumor cell death (14). Tumor-infiltrating macrophages are plentiful within the tumor microenvironment and regulate the activity of T cells (15), tumor-associated macrophages and T cells play a key role in determining cancer prognosis and the efficacy of immunotherapies (16). Whereas macrophages and T cells were found to be statistically significant in the present study, therefore, the immunological profile of TME in osteosarcoma is considered to have prognostic value.

The results showed that ImmuneScore had a significant correlation with prognosis, with higher ImmuneScore representing a higher level of immune infiltration, leading to higher survival. TumorPurity was inversely correlated, with high TumorPurity associated with low survival. Subsequently, we analyzed the correlation between the mutations and immunological features of the genes. The correlation between mutations in the TP53, ATRX, and RB1 genes and osteosarcoma has been extensively studied. TP53 prevents the transformation of bone marrow mesenchymal stem cells to osteosarcoma (17), and Lang et al. showed that (18), in mice, mutation of TP53 promotes the development of osteosarcoma. Furthermore. Walkley et al. showed (19) that combined deletion of TP53 and RB1 in mouse osteoblasts leads to a high frequency of metastatic osteosarcoma, and that mutations in RB1 are a key driver of cancer (including osteosarcoma) (20). A meta-analysis (21) that included 491 patients with osteosarcoma showed that RB1 mutations were associated with a significantly reduced histological response to chemotherapy and a high risk of metastasis in osteosarcoma. ATRX plays an important tumor-suppressor role in OS, and deletion of this gene leads to tumor cell growth, migration, and invasion, and was one of the most commonly mutated genes in 288 osteosarcoma patients surveyed by the Genomics Evidence Neoplasia Information Exchange consortium in the USA (22). Taken together, mutations in the TP53, ATRX, and RB1 genes promote the development, invasion, and metastasis of osteosarcoma, and in **Figure 3**, the mutation frequency of these three genes is significantly higher in the low-immunity group than in the high-immunity group, indicating that the frequency of metastasis of osteosarcoma is also higher in the low-immunity group, which is in line with the results we obtained above.

Based on the findings from GO, KEGG, and GSEA enrichment analyses, numerous differentially expressed genes (DEGs) showed enrichment in pathways related to the immune system. Neutrophil chemotaxis was associated with more DEGs during BP in all GOs. Previous studies have shown that neutrophils are a major component of TME (23), which can exert a tumour-killing effect by affecting T cells (24, 25). In this study, two groups with high and low levels of immune activation were also studied in depth, and noticeable variances were observed in the extent of immune cell infiltration between the two groups, and the level of immune cell infiltration in the high-immunity group was significantly higher than that in the low-immunity group, which may be associated with the high survival rate of the high-immunity group. Ten pivotal genes were then identified, all of which are closely associated with neutrophils, macrophages, and T cells.

Following that, a univariate COX regression analysis and LASSO analysis were utilized to identify PDK1 and PPARG. A risk model was then established using these two factors, revealing a pronounced prognostic distinction between individuals in the high-risk and low-risk categories. Notably, the low-risk group exhibited a significantly superior prognostic survival rate. Pyruvate dehydrogenase kinase-1 (PDK1) is an enzyme involved in glycolysis that facilitates the transition from glucose oxidative metabolism to glycolytic metabolism in cancer cells by phosphorylating substrates (26) and also reduces the damage caused by reactive oxygen species (ROS) accumulation. In recent years, more and more evidence suggests that PDK1 is associated with tumor progression and metastasis (27–29), which provides a new idea for the development of targeting PDK1 for the treatment of osteosarcoma, as evidenced by Liu et al. who constructed a novel organoarsenic compound, Aa-Z2, which induces apoptosis of osteosarcoma by reprogramming metabolism through targeting PDK1 (30). Peroxisome proliferator-activated receptor-gamma (PPARG), a member of the nuclear receptor family, is a major regulator of adipocyte differentiation and function (31). PPARG has been shown to play a role in several cancers, and its association with cancer is primarily a result of the recording of PPARG in cancer cells and the tumor cell microenvironmental role (32). The role of PPARG is widely debated and it exerts inhibitory or promotional effects on cancer growth depending on the tumor cell conditions and the pathways stimulated (33). In the literature, PPARG is an oncogene, which exerts anti-tumour effects by inhibiting cell proliferation, differentiation, cell growth, cell cycle, and inducing apoptosis. It has been shown that in human osteosarcoma, the pro-apoptotic effects exerted by Oridonin inhibition of the Nrf2 pathway require PPARG activation (34). PPARG can trigger cell apoptosis and suppress the growth of osteosarcoma cells by facilitating the terminal differentiation of osteoblasts (35).

When constructing the risk model, it was found that elevated levels of PDK1 were linked to the high-risk score group, which in turn was correlated with increased mortality rates. This implies that PDK1 may act as an oncogene. In contrast, high expression of PPARG was positively associated with the low-risk score group, which played the role of oncogene, which was consistent with the findings of previous scholars mentioned above, and further proved the accuracy of the risk model. While the high expression of PDK1 is proportional to the high expression of Macrophages M0 and inversely proportional to the high expression of Macrophages M2, the opposite is true in PPARG. Lin et al. Showed (36) that Nuanxinkang (NXK) reduced the transcript and protein levels of HIF-1α and PDK1 *in vivo*. NXK inhibited macrophage M1 and significantly increased macrophage M2 via the HIF-1α/PDK1 axis, and PDK1 and macrophage M2 levels *in vivo* were negatively correlated, which is in line with the findings of this study. Consistent with the findings of this study. Macrophage M1 is biased towards glycolytic metabolic processes, whereas macrophage M2 is more biased towards oxidative phosphorylation (OXPHOS) metabolic processes, and under the stimulation of lipopolysaccharides, the macrophage shifts from OXPHOS metabolism to glycolytic metabolism, PDK1 is a glycolytic enzyme, and when PDK1 is inhibited, glycolytic metabolism is inhibited, and the oxidative phosphorylation metabolic process is also strengthened, thus promoting the macrophage M2. PPARG agonists promote macrophage M2 polarisation (37). When PPARG signaling is inhibited, it promotes the macrophage transition from M2 to M1 (38), suggesting a positive correlation between PPARG levels and macrophage M2 levels, validating the accuracy of the findings of this study. Tumor-associated macrophages (TAM) are populations of macrophages that infiltrate into tumor tissue, including the M1 and M2 cell populations. TAM is closely associated with tumors, with M1 acting as an anti-tumor agent and M2 inhibiting T cell-mediated anti-tumor effects and promoting tumor formation (39, 40). TAMs are derived from monocytes in the bone marrow, and a variety of cytokines and chemokines can direct the migration of monocytes to the tumor site (41), the growth of tumors can also result in the transformation of CCR2+ monocytes into TAMs (42). TAMs can modulate the cytotoxicity of T cells and NK cells towards tumor cells. TAM can suppress the proliferation of CD8 T cells by nitrogen species, iNOS, and oxygen radicals (43–45). In addition, TAM can further inhibit the antitumor effects produced by T cells by recruiting Treg cells via CCL22 (46). Chen et al.’s study (47) showed that TAM promotes tumor growth by generating inflammatory Th subpopulations to stimulate an inflammatory response in tumors. TAM is also regulated by other immune cells, and Treg cells function to inhibit the release of IFN-γ from CD8 T cells (48), which is the main cytokine responsible for macrophage M2 inhibition; thus, Treg indirectly and selectively maintains metabolic fitness and survival of M2-like TAM. A study by Kumar et al. (49) showed that myeloid-derived suppressor cells (MDSC) could regulate TAM differentiation by down-regulating STAT3, promoting tumor proliferation. In addition, B cells can also induce macrophage M2 polarisation in tumors and inhibit T cells and macrophage M1 from promoting tumor proliferation (50). The role of Macrophages M0 for osteosarcoma is currently unclear (51). Therefore, immunotherapy targeting macrophage transformation may become a promising therapeutic strategy for the treatment of osteosarcoma.

In recent years, immunotherapy has been a widely researched therapeutic approach that has achieved excellent results in the treatment of many types of cancer. Immune checkpoint inhibitors (ICIs) are a form of immunotherapy that works by stimulating the body’s immune system to combat cancer. This is achieved through the inhibition of immune checkpoint molecules like programmed cell death-1 (PD-1) and programmed cell death ligand-1 (PD-L1), which play crucial roles in regulating the immune response. programmed cell death ligand-1 (PD-L1) to activate the body’s immune response to fight cancer. The results have been satisfactory in the treatment of many cancers. Nevertheless, targeted PD-1/PD-L1 therapy yields unsatisfactory outcomes in osteosarcoma (52), possibly attributed to the distinct PD-1/PD-L1 regulation in the tumor, commonly known as a “cold tumor” (53). Numerous studies have indicated a relationship between elevated levels of PD-L1 and unfavorable outcomes in osteosarcoma patients, yet the precise role of PD-L1 in osteosarcoma pathogenesis remains ambiguous. It can be seen from this study that most of the immune checkpoint-related genes have higher gene expression in the low-risk scoring group and lower gene expression in the high-risk scoring group, which suggests that the risk model obtained in this study has significance for the gene regulation of immune checkpoints. High expression of these immune checkpoint-associated genes was associated with better prognosis, whereas PDK1 was highly expressed in the high-risk scores and was associated with poorer prognosis, so PDK1 was negatively correlated with the expression of immune checkpoint-associated genes, whereas PPARG was highly expressed in the low-risk scores group, and, in contrast to PDK1, PPARG was positively correlated with the expression of immune checkpoint-associated genes. This may be because the tumor microenvironment in the low-risk group was more amenable to immune cell infiltration and activation, resulting in increased expression of immune checkpoint molecules, reflecting good immune surveillance of the tumor. High expression of immune checkpoint genes is associated with a better clinical prognosis, and we speculate that this may be because the immune system of osteosarcoma patients can efficiently recognize the tumor and develop an immune response, and because highly mutated genes in osteosarcoma does not produce sufficient neoantigens that can elicit an immune response so that targeted inhibition of PD-1/PD-L1 therapy in osteosarcoma is unsatisfactory. Therefore, PDK1 and PPARG may become prognostic genes in osteosarcoma and may be targets for subsequent regulation of ICI-related genes for osteosarcoma treatment.

As we said above, mutations in TP53, RB1, and ATRX genes can promote the growth, invasion, and metastasis of osteosarcoma, which is a kind of ‘cold tumor’, and the microenvironment of osteosarcoma can monitor the tumor well, and it is less responsive to immune checkpoint blockade, which is confirmed by the calculation of the TIDE score, so the effect of high TMB on osteosarcoma is more inclined to promote the development of osteosarcoma, resulting in a poorer prognosis of osteosarcoma patients with high TMB.

In this study, the majority of IR-DEGs were found to be overrepresented in T lymphocytes. It has been well-documented in previous research that the infiltration and activation of T cells are crucial in the therapeutic management of osteosarcoma, and that adoptive T-cell therapy (ACT) has a promising future for the treatment of osteosarcoma, whereas ICI activates the immune system, ACT directly “tells” the T-cells the characteristics of the tumor, and then attacks the tumor in a targeted manner. Based on prior studies, it has been recognized that there are three primary categories of penicillin combination treatments: chimeric antigen receptor (CAR)-modified T cells, T cell receptor (TCR)-modified T cells, and tumor-infiltrating lymphocytes (TILs) (54). Our research findings indicate that there was a notable difference in the extent of immune cell infiltration between the hyperimmune and hyperimmune groups, with a marked increase in the hyperimmune group. This heightened immune response was correlated with a better survival outcome in the hyperimmune group. Ten hub genes were obtained in this study, which were significantly correlated with TILs and therefore they are highly specific for targeting tumors. Combining ICI with TIL T-cells may also be an effective option for individual therapy, and recent findings by Wang et al. showed that TILs in combination with anti-PD1 therapy demonstrated significant clinical efficacy in patients with metastatic osteosarcoma compared to anti-PD1 therapy applied alone. The objective remission rate of this combination regimen was almost five times higher than that of single anti-PD1 therapy, while intermediate progression-free survival and intermediate overall survival were also significantly prolonged (55).

More and more studies are being conducted, and the present study fully considers the effect of immune infiltration on osteosarcoma and uses it for risk modeling, demonstrating excellent prognostic specificity and providing a novel and valuable tool for future research.

## 5 Strengths and limitations

The strength of this study is the use of bioinformatics to investigate osteosarcoma from the perspective of immune infiltration, which revealed that higher immune infiltration has a better prognosis, and then concluded that two important genes, PDK1 and PPARG, whose high or low expression is associated with the prognosis of osteosarcoma, and whose effect on Macrophages M0 and Macrophages M2 regulation also has a crucial impact and can even regulate immune checkpoint-related genes. Subsequently, a risk model was constructed using PDK1 and PPARG, and the risk model provided a good prognosis prediction.

This study has some limitations. This study only used computers and their related software to analyze the data, and it still lacks relevant experimental validation. In our future work, we will further expand the clinical samples and conduct animal or human experiments to improve the study’s accuracy and lay a more solid foundation for treating osteosarcoma.

## 6 Conclusions

From the above description of this study, it can be concluded that a high Immune score is associated with a better prognosis in osteosarcoma. Subsequently, several analyses were performed to verify the effect of immune infiltration on osteosarcoma, firstly, the samples were immuno-infiltrated using ssGSEA, and the samples were divided into two groups based on the immune score, with the group with high immune activation having a significant survival advantage over the other group. Then, using a univariate COX regression analysis and LASSO analyses, two genes, PDK1 and PPARG, were obtained, and a risk model was constructed based on the derived genes, in which PDK1 was positively correlated with the risk score, and PPARG was negatively correlated with the risk score, and through further analyses, we found that PDK1 was negatively correlated with macrophage M2, and the opposite was true for PPARG, and that the group with a high-risk score had a more high TMB and their prognosis was poorer. We also analyzed immune checkpoint-related genes, which were negatively correlated with risk scores, suggesting that the osteosarcoma microenvironment has good tumor surveillance and responds poorly to ICB treatment. Finally, we also analyzed the TMB of the samples. We found that high TMB was associated with low immune infiltration and that an increased mutation rate increased the risk of osteosarcoma. Therefore, the prognostic model obtained in this study is suitable for further optimization and eventual clinical application.

## Data Availability

The original contributions presented in the study are included in the article/**Supplementary Files**, further inquiries can be directed to the corresponding author/s.
